# Complement Inhibitors for Advanced Dry Age-Related Macular Degeneration (Geographic Atrophy): Some Light at the End of the Tunnel?

**DOI:** 10.3390/jcm12155131

**Published:** 2023-08-04

**Authors:** Miguel Cruz-Pimentel, Lihteh Wu

**Affiliations:** 1 Department of Ophthalmology and Vision Sciences, University of Toronto, Toronto, ON M5S 1A1, Canada; hes.oftalmologia.mcp@gmail.com; 2 Asociados de Macula, Vitreo y Retina de Costa Rica, Primer Piso Torre Mercedes Paseo Colon, San José 10102, Costa Rica; 3 Illinois Eye and Ear Infirmary, Department of Ophthalmology, School of Medicine, University of Illinois Chicago, Chicago, IL 60612, USA

**Keywords:** age-related macular degeneration, geographic atrophy, complement inhibitors, pegcetacoplan, avacincaptad, C3, C5, iRORA, cRORA

## Abstract

Geographic atrophy (GA) affects around 5 million individuals worldwide. Genome-wide, histopathologic, in vitro and animal studies have implicated the activation of the complement system and chronic local inflammation in the pathogenesis of GA. Recently, clinical trials have demonstrated that an intravitreal injection of pegcetacoplan, a C3 inhibitor, and avacincaptad pegol, a C5 inhibitor, both statistically significantly reduce the growth of GA up to 20% in a dose-dependent fashion. Furthermore, the protective effect of both pegcetacoplan and avacincaptad appear to increase with time. However, despite these anatomic outcomes, visual function has not improved as these drugs appear to only slow down the degenerative process. Unexpected adverse events included conversion to exudative NV-AMD with both drugs. Occlusive retinal vasculitis and anterior ischemic optic neuropathy have been reported in pegcetacoplan-treated eyes.

## 1. Introduction

Age-related macular degeneration (AMD) is a significant cause of blindness, representing 8.7% of all blindness cases worldwide. The projections show that its incidence will increase by 2040 and may affect up to 400 million people [1]. Late AMD has two components, a neovascular (NV) and a non-neovascular component. The advanced late stage of the non-NV component is called geographic atrophy (GA) [2]. GA affects around 5 million individuals worldwide [1,3,4].

**Figure 1:**
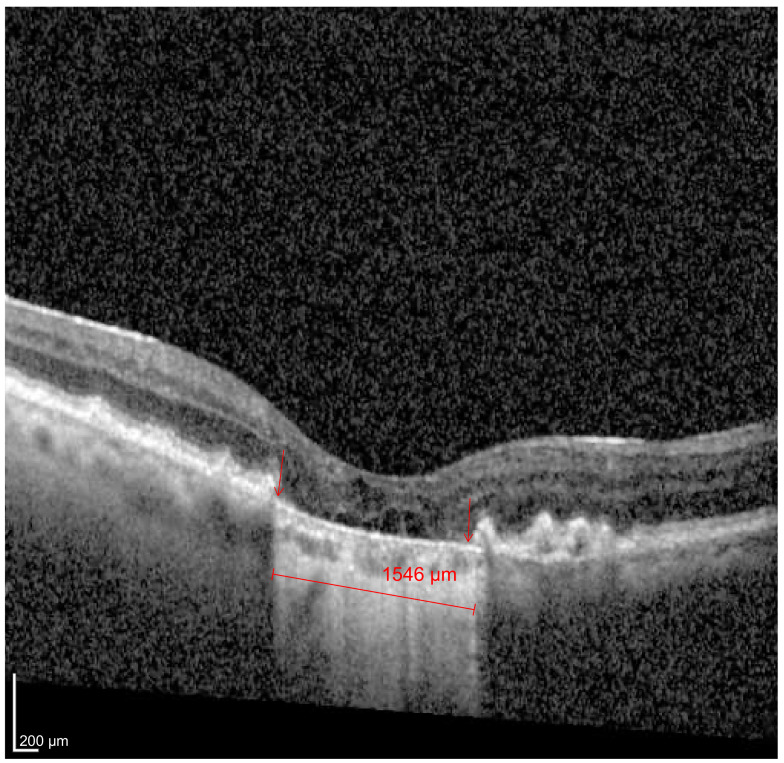
Complete retinal pigment epithelium outer retinal atrophy (cRORA). Foveal cut of an SD-OCT (Heidelberg Spectralis) image of an 89-year-old woman with a visual acuity of 20/400. Both the areas of hypertransmission and RPE (area between the red arrows) match and measure 1546 µm.

GA is characterized by an insidious and progressive loss of photoreceptors, retinal pigment epithelium (RPE) and the choriocapillaris. It typically starts affecting the perifoveal region and spares the central fovea until the very end [4,5]. Recognizing spectral-domain optical coherence tomography’s (SD-OCT) ability to discriminate the different macular anatomic layers and its widespread availability in the daily management of AMD, recently, the Classification of Atrophy Meeting Group (CAM) [6] defined incomplete retinal pigment epithelium outer retinal atrophy (iRORA) and complete retinal pigment epithelium outer retinal atrophy (cRORA) in the hopes of better characterizing GA. These definitions were based on the extension of the RPE and outer retina loss as seen with SD-OCT [7]. cRORA was defined as (1) a region of hypertransmission of at least 250 μm in diameter; (2) a zone of attenuation or disruption of the RPE of at least 250 μm in diameter; (3) evidence of overlying photoreceptor degeneration, whose features include outer nuclear layer (ONL) thinning, external limiting membrane (ELM) loss and ellipsoid zone (EZ) or the interdigitation zone (IZ) loss; and (4) the absence of scrolled retinal pigment epithelium (RPE) or other signs of an RPE tear [7] (Figure 1).

**Figure 2:**
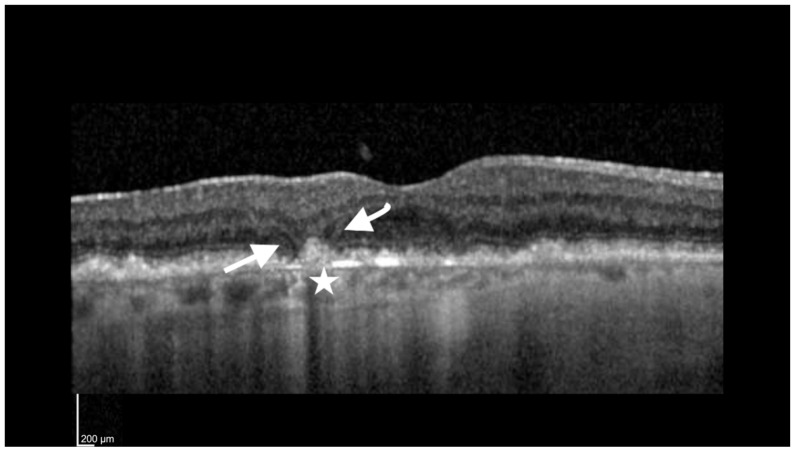
Incomplete retinal pigment epithelium outer retinal atrophy (iRORA). Foveal cut of an SD-OCT (Heidelberg Spectralis) image of an 83-year-old woman with a visual acuity of 20/80. The arrows point to the presence of a hyporeflective wedge in the Henle fiber layer (HFL). The star is adjacent to an area of choroidal hypertransmission <250 µm.

iRORA was defined by the following criteria: (1) a region of signal hypertransmission into the choroid, and (2) a corresponding zone of attenuation or disruption of the RPE, with or without the persistence of basal laminar deposits (BLamD) and evidence of overlying photoreceptor degeneration, i.e., the subsidence of the inner nuclear layer and outer plexiform layer (OPL), presence of a hyporeflective wedge in the Henle fiber layer (HFL), thinning of the ONL, disruption of the ELM or disintegrity of the EZ, and when these criteria did not meet the definition of cRORA (Figure 2).

The pathophysiologic mechanisms involved in the initiation and progression of GA remain poorly understood. Several clinical trials have tested different drugs with different mechanisms of action including brimonidine, ciliary neurotrophic factor delivered by encapsulated cell therapy, an antiamyloid beta monoclonal antibody and visual-cycle modulators such as fenretinide and emixustat, among others. None of these approaches have yielded positive results [8,9,10,11].

Genome-wide, histopathologic, in vitro and animal studies have implicated the activation of the complement system and chronic local inflammation in the pathogenesis of GA [12,13,14,15,16]. The complement system is composed of more than 30 proteins in the plasma that are part of the innate immune system. The three main functions of the complement system are defense against infection, bridging the adaptive and innate immune system and the disposal of waste by mediating the clearance of immune complexes and apoptotic cells [17]. The complement system can be activated via three pathways, namely the classical pathway, the lectin pathway and the alternative pathway. Each of these pathways have different activators [17]. For instance, antigen–antibody complexes are bound by circulating C1q activating the classical pathway. Pathogens that express mannan binding lectin on their surface lead to the activation of the lectin pathway. The alternative pathway appears to be in a constant low state of activation where C3 is spontaneously hydrolyzed into C3a and C3b. The alternative pathway may be amplified via a feedback loop. All the above pathways converge upon a common terminal pathway that sequentially assembles C5b, C6, C7, C8 and C9, culminating in the formation of the membrane attack complex (MAC). The MAC promotes cell lysis by forming pores across the cellular bilipid layer [4,17,18,19].

The main sources of complement components and many circulating complement regulatory proteins are liver hepatocytes, which release these proteins into the bloodstream. However, in certain tissues where there is limited access to these circulating proteins, the machinery to synthesize complement extrahepatically exists. A quantitative polymerase chain reaction analysis revealed that the cells in the human RPE–choroid complex express a complete set of transcripts associated with both the alternative and classical complement pathways. In contrast, with the exception of C5 and C7, the other components of the lectin and terminal pathway relies on the systemic circulation to be delivered to the human RPE–choroid complex.

Under normal conditions, complement-related gene expression is limited to the terminal and inhibitors of the alternative pathways [16]. In susceptible individuals that carry risk variants of complement components, the expression of these variants in the RPE may lead to dysregulation and over-reactivity in the complement cascade. These events can promote AMD by several mechanisms. For instance, drusen represent the hallmark of early and intermediate AMD. They are extracellular deposits composed of complement activators, complement regulatory proteins and complement factors. This suggests that a chronic local inflammatory and immune-mediated event at the level of the RPE–Bruch’s membrane complex may play a central role in drusen biogenesis [16]. Hyperactivity in the MAC complex may promote the lysis of cells in the RPE, choriocapillaris and photoreceptors [20]. The complement fragments C3a and C5b direct macrophages and microglia cells into the subretinal space, promoting inflammation [20]. The Y402H variant of factor H can promote the accumulation of phagocytes, leading to inflammation in the subretinal space and the RPE cells [20,21]. Dysregulation in the complement cascade can lead to NLPR3 inflammasome activation. The NLPR3 inflammasome is a group of proteins composed of NLPR3, the adaptor molecule ASC and caspase 1. Once activated, this system can promote the secretion of the cytokines interleukin-1β and IL-18, which then lead to a specific type of cell lytic death called pyroptosis [4,20,22].

Recently, clinical trials have demonstrated a reduction in the growth of GA following the inhibition of C3 and C5. The United States Food and Drug Administration (USFDA) has recently approved pegcetacoplan (Sifovre, Apellis Pharmaceuticals Inc., Waltham, MA, USA), a C3 inhibitor, for the treatment of GA. Avacincaptad pegol (Zimura, Iveric Bio, Parsippany, NJ, USA), a C5 inhibitor, has been granted a Fast Track designation by the USFDA. The purpose of the current manuscript is to review the inhibition of the complement factors C3 and C5 in patients with GA.

## 2. Pegcetacoplan (Sifovre)

Pegcetacoplan binds to C3 and C3b and regulates the overactive complement system. It consists of two copies of a tridecapeptide that are covalently conjugated to a linear polyethylene glycol molecule through a Lys linker to enhance its half life [18,23]. The phase 2 FILLY study included 246 patients with GA [24]. The diagnosis of GA had to be confirmed by blue fundus autofluorescence (FAF) with an area of atrophy ≥2.5 mm^2^ and ≤17.5 mm^2^. In addition, any hyperautofluorescence in the junctional zone of the GA area had to be present. Eyes with multifocal lesions had to have at least one lesion ≥1.25 mm^2^ [24]. The patients were randomized to monthly injections or injections every two months (EOM) or sham intravitreal injections of 15 mg of pegcetacoplan [24].

The growth of GA is dependent on the baseline area of GA [25]. The square root transformation of the GA lesion area corrects for this dependency [26]. The primary outcome of the FILLY trial was the 12-month change in the square root transformed area of the atrophy extension from baseline as measured by FAF. A statistically significant reduction in the growth rate of the GA square root transformed area was seen in both the monthly (29%) and the EOM (20%) arms when compared to the control arm. This beneficial effect was most pronounced between months 6 and 12 of the treatment, where the reduction in the growth rate was 45% and 33% in the monthly and EOM arms, respectively. Conversely, if the treatment was stopped at month 12, the effect of reducing the extent of the GA growth was significantly reduced [24]. The FILLY study identified both an extrafoveal GA lesion and a larger low luminance deficit (LLD) as independent risk factors of GA progression. It also showed that after correcting for these factors, the treatment effect was maintained [27]. Intravitreal pegcetacoplan was also able to significantly reduce photoreceptor loss and thinning, which was assessed by a fully automated deep learning algorithm that segmented the RPE and photoreceptors in SD-OCT volume scans [28]. Pegcetacoplan also significantly reduced the progression of iRORA to cRORA. At a one-year follow up, iRORA progressed to cRORA in 50.0% of the monthly arms, 60% of the EOM arms and 82% of the arms in the sham group. These results suggest that pegcetacoplan may be beneficial in earlier stages of AMD [29]. Pegcetacoplan did not affect foveal encroachment by GA during the 12 months of treatment in this study [24].

Even though the primary endpoint of the pegcetacoplan trials were based on FAF imaging, FAF has certain shortcomings that need to be stated. FAF identifies RPE atrophy, but it does not identify photoreceptor loss and it does not assess the status of the junctional zone, nor does it reliably identify RPE atrophy within the fovea. In contrast, SD-OCT can reliably overcome these FAF shortcomings [28,30]. Mai et al. [30] compared and correlated the FAF and the SD-OCT outcomes of the FILLY trial. SD-OCT was found to be as reliable as FAF at determining GA lesion growth. In addition, SD-OCT was able to assess EZ impairment, leading the authors to conclude that SD-OCT could be a more sensitive monitoring tool during GA treatment [30]. Another post hoc analysis of the FILLY trial analyzed the SD-OCT images at baseline, 2 months, 6 months and 12 months. Deep learning automated segmentation of the RPE and photoreceptor thickness was performed. Intravitreal pegcetacoplan led to a reduction in photoreceptor loss and thinning when compared to the sham injections. These results provide proof of principle that intravitreal C3 inhibition can preserve photoreceptors [28].

In another post hoc analysis of the FILLY trial, Vogl et al. [31] used deep learning algorithms to identify disease activity and the effects of pegcetacoplan on the progression of GA. The GA lesions on the Heidelberg Spectralis SD-OCT were automatically segmented. The local progression rate was calculated by using a growth model that measured the local growth of the GA lesion margins. Furthermore, they also looked at the mean photoreceptor thickness, hyper-reflective foci concentration and the direction of the GA growth at each individual point on the GA lesion margin. They found that the local progression rate was slower in areas with thicker photoreceptor layers and lower hyper-reflective foci concentrations. For lesions that are actively growing towards the fovea, the closer they get to the center of the fovea, the more the growth slows. There is a peak progression rate at 1 mm eccentricity to the fovea. These researchers confirmed that pegcetacoplan was able to significantly slow down the local progression rate of GA [31].

Outcomes related to visual acuity and the quality of vision did not demonstrate any beneficial effect of pegcetacoplan. All three groups showed a gradual decline in vision, low-luminance BCVA and LLD without any significant differences between the three groups [24,32,33].

The pivotal phase 3 clinical trials OAKS and DERBY enrolled 637 and 621 patients, respectively [32]. Both studies had the same design, and their inclusion criteria were similar to those from FILLY. The primary outcome measured was the growth of the GA area from baseline to month 12. Pegcetacoplan significantly reduced the growth of GA by 21% in the monthly arms and 16% in the EOM arms in the OAKS trial. In contrast, the primary outcome was not achieved in DERBY since the reductions obtained were only 12% and 11% for the monthly and EOM arms, respectively [32]. As time went on, the percent reduction in GA growth grew in the pegcetacoplan-treated eyes as compared to the sham-treated eyes. At 24 months, a reduction in GA growth of 36% was achieved in the group receiving monthly injections and 29% in the EOM arms of the DERBY trial compared to a 24% reduction in the monthly arms and 25% in the EOM arms in OAKS [33].

Given the important role that the complement system plays in fighting infection, one of the major concerns of using complement inhibitors is the theoretical risk of an increase in infections. In the FILLY trial, culture-positive infectious endophthalmitis was reported in 2.3% of the eyes in the monthly group compared to 0% in the sham group. In the EOM group, there was a single case (1/79 = 1.3%) of endophthalmitis, which happened to be culture-negative [24]. The 24-month combined incidence of endophthalmitis from OAKS and DERBY was 0.5% in both the monthly and EOM groups. The combined rate of ocular inflammation was 3.8% in the monthly injections group and 2.1% in the EOM group. No cases of retinal vasculitis were reported [33]. However, on 15 July 2023, the American Society of Retinal Specialists (ASRS) Research and Safety in Therapeutics (REST) Committee warned its members that six patients had developed occlusive retinal vasculitis following an administration of pegcetacoplan. These occurred between 7 and 13 days after the pegcetacoplan injection. Up until then, approximately 60,000 vials of pegcetacoplan had been distributed. No specific lots were identified (https://www.asrs.org/clinical/clinical-updates/9327/ASRS-Research-and-Safety-in-Therapeutics-REST-Committee-Update-on-Adverse-Events, accessed on 22 July 2023).

An unexpected dose-dependent increased rate of the new onset exudative NV-AMD was observed in the eyes treated with pegcetacoplan when compared to the sham-treated eyes. In the FILLY study, the eyes treated with pegcetacoplan monthly converted 20.9% of the time vs. 8.9% in the EOM eyes vs. 1% in the sham-treated eyes [24]. In the OAKS and DERBY studies, the conversion rates to exudative NV-AMD were much lower at 6.0%, 4.1% and 2.4% in the monthly, EOM and sham-injection groups, respectively [32]. These rates increased to 11.9%, 6.7% and 3.1% in the monthly, EOM and sham group, respectively, at 24 months [33]. The risk factors for the development of macular neovascularization (MNV) included MNV in the fellow eye at baseline and the presence of the double-layer sign (DLS) during SD-OCT at baseline [34]. The DLS consists of two highly reflective layers, the RPE and another highly reflective layer beneath the RPE, typically found in areas of the branching vascular network seen in polypoidal choroidal vasculopathy (PCV) [35]. However, the DLS is not pathognomonic of PCV. Other studies found a high correlation between the presence of the DLS and the presence of type 1 MNV or nonexudative neovascular AMD [36,37]. The mechanism of exudative MNV following complement inhibition remains unclear.

Ischemic optic neuropathy (ION) was reported in 1.7%, 0.2% and 0% of eyes in the pegcetacoplan monthly, EOM and sham groups, respectively. All of these eyes had discs at risk and multiple systemic risk factors. The underlying mechanism behind the presentation of ION has yet to be determined [33].

## 3. Avacincaptad Pegol (Zimura)

Eculizumab is a murine humanized monoclonal antibody that targets C5. C5 inhibition potentially preserves the anti-inflammatory properties of C3a. It has been approved for the treatment of paroxysmal nocturnal hemoglobinuria and atypical hemolytic uremic syndrome. Patients underwent systemic treatment with eculizumab via an intravenous infusion of 600 mg to 1200 mg weekly for 24 weeks. Systemic eculizumab did not slow down the growth of GA [38].

Avacincaptad pegol is a pegylated RNA aptamer with a high affinity to complement factor C5 [39]. The GATHER1 study was a phase 2/3 study that consisted of two parts. In part 1, 77 patients were randomized to receive 1 mg of avacincaptad or 2 mg of avacincaptad or sham monthly intravitreal injections. In part 2, 209 patients were randomized to receive 2 mg of avacincaptad or 4 mg of avacincaptad or sham monthly intravitreal injections. The 4 mg dose of avacincaptad was delivered by two intravitreal injections of 2 mg [40]. Eligible patients had to have GA without the involvement of the foveal center, and it had to be located within an area of 1500 mm from the center of the fovea. The total area of GA had to be between 2.5 and 17.5 mm^2^, as determined by blue FAF imaging. Patients with multifocal GA were also included if they had at least one focal lesion of ≥1.25 mm^2^ [39,40].

As in the pegcetacoplan studies, square root transformations of the GA lesion area were performed to compare the growth rates of the GA lesion size. At a 12-month follow up, the monthly 2 mg arm had a 27.4% reduction in GA growth compared to the sham group. The 4 mg monthly arm had a similar reduction in GA growth of 27.8% when compared with the sham group at month 12 [39]. The earliest growth-reducing effect of GA was seen at 6 months, with a 28.4% reduction for the 2 mg injection arm and a 26.6% reduction for the 4 mg arm [39]. At an 18-month follow up, there was a further reduction in the growth in both treatment arms with respect to the sham arm. The 2 mg and 4 mg arms had a 28.1% and 30% reduction in the GA lesion size, respectively [40].

Based on the favorable results of the GATHER1 study, the confirmatory phase 3 study GATHER2 was designed and executed. In GATHER2, 447 patients participated and were randomized to receive 2 mg of avacincaptad and sham injections [41]. A total of 2 mg of Avacincaptad was used since the efficacy of the 2 and 4 mg was similar and the safety profile was better in the 2 mg arm [40]. The inclusion criteria were identical to GATHER1. At 12 months, a statistically significant reduction in the mean rate of the GA growth of 14.3% by using a square root transformation was reported in the group treated with 2 mg of avacincaptad versus the sham-treated group [41].

Similar to pegcetacoplan, in the GATHER1 study, the avacincaptad-treated eyes had a higher risk of converting to exudative NV-AMD. Exudative NV-AMD was diagnosed by clinical examination, SD-OCT or fluorescein angiography. OCT angiography was not available. At a 12-month follow up, 9.0% of the eyes in the 2 mg cohort and 9.6% of the eyes in the 4 mg cohort developed MNV [39]. By 18 months, a conversion was observed in 11.9%, 15.7% and 2.6% of cases in the 2 mg, 4 mg and sham group, respectively. The fellow eyes converted from 3% to 3.6%. Unfortunately, the patients that developed MNV were exited from the study; therefore, the details of the clinical course and impact on BCVA are limited [40]. In GATHER2, at 12 months, 6.7% and 4.1% of the avacincaptad- and sham-treated eyes developed MNV. In GATHER2, there were no cases of intraocular inflammation, endophthalmitis or ION [42].

The Supplementary Table S1 summarizes the DERBY, OAKS, GATHER1 and GATHER2 studies.

## 4. Conclusions and Future Directions

Since treatment options are now available for patients with GA, a clear distinction should be made between atrophy that is secondary to an inherited retinal disease and atrophy that is secondary to AMD [43]. Multimodal imaging is particularly useful when making this distinction. For instance, end-stage Stargardt disease may easily be confused with GA secondary to AMD. Both OCTA and indocyanine green angiography (ICG-A) may differentiate between these two conditions [44,45]. The areas of atrophy typically manifest ICG-A hypocyanescence whereas atrophic areas in AMD typically manifest iso or mild hypercyanescence [44]. OCT-A imaging showed that eyes with macular atrophy secondary to Stargardt disease had choriocapillaris loss in the center with persistent tissue at the margins. In contrast, in the AMD eyes, the choriocapillaris was present but rarified [45].

Despite the success shown by both C3 and C5 inhibitors delivered intravitreally in slowing the progression of GA, many challenges still lie ahead. Both have demonstrated dose-dependent reductions in the rate of growth of GA. A post hoc analysis of the FILLY trial showed that pegcetacoplan reduced the rate of progression from iRORA to cRORA, suggesting that it may be beneficial in earlier stages of AMD [29]. The protective effect of both pegcetacoplan and avacincaptad appear to increase with time. However, despite these anatomic outcomes, visual function has not improved as these drugs appear to only slow down the degenerative process [24,33,39,40,41]. Patients that opt to be treated by any of these drugs will soon realize that there is a very high treatment burden of injections with no apparent benefit perceived by them. The key questions remaining involve the timing of the initiation of the treatment in an individual patient. How early should we intervene to avoid the development of irreversible damage? Would the outcomes be the same if patients are treated earlier? Unfortunately, we do not currently have the answers to these and many other questions.

GA lesions are very heterogeneous and vary particularly in their growth patterns. Reported GA growth rates vary anywhere from 0.53 to 2.6 mm^2^ per year [46]. There are many factors that influence the direction of growth (towards the fovea vs. towards the periphery) and the velocity of growth. The FAF pattern in the junctional zone of the GA lesion may predict the rates of progression of GA. Eyes with banded or diffuse hyper FAF patterns demonstrated faster growth rates when compared to eyes without hyper FAF patterns or with just focal hyper FAF patterns [47]. Others have reported that the presence of reticular pseudodrusen (subretinal drusenoid deposits) accelerates the growth of GA [48,49,50]. Lesion size also determines growth rates. Smaller lesions tend to grow slower whereas larger lesions tend to grow faster. Extrafoveal and multifocal lesions also grow faster than foveal and unifocal lesions [46,51]. In patients whose fellow eye also harbors a GA lesion, the GA lesion grows faster [52]. Choriocapillaris flow deficits and impairment may also affect the growth rates of GA lesions [53,54]. Patient ethnicity may also play a role. A recent study highlighted the differences in GA lesion phenotype, growth rate and associated features in Asians compared to non-Asians [55]. Asian patients exhibited thicker choroids, fewer drusen and smaller GA lesions with fewer GA foci compared to non-Asian patients. The proportion of eyes with a diffuse or banded FAF pattern was similar between both groups. In general, Asian eyes with GA had a slower lesion growth than non-Asian eyes [55]. Ideally, risk stratification should help in decision making when deciding to consider initiating therapy with these novel drugs. Before we can use risk calculators that take into account the direction of growth, location and predicted growth rate as suggested by Guymer, much work still needs to be conducted. In order to accomplish this, a recent editorial by Guymer [43] emphasizes the need to image all of our GA patients with FAF and SD-OCT. Artificial intelligence algorithms may then use these images to help the decision-making process.

A better understanding of the role of the complement system in the pathogenesis of AMD is definitely needed. A genetic analysis of 47 genes that included CFH, CFI, C2/CFB and C3 was undertaken in the FILLY trial. None of these genes influenced the response to treatment with pegcetacoplan. Two genetic factors, rs2230199 in C3 and rs3750846 in ARMS2, were found to influence the rate of growth of GA, regardless of the treatment group to which the patients were assigned. This indicates that pegcetacoplan slows GA progression independent of these genetic risk factors [24]. Furthermore, a recent post hoc analysis on aqueous humor and plasma samples of 81 patients from the Chroma and Spectri trials showed that the complement levels or activities in the aqueous humor or plasma did not correlate with the GA lesion size or growth rate [56].

A significant concern with these complement inhibitors is the conversion to exudative NV-AMD, which appears to be dose dependent with both C3 and C5 inhibition [34,42]. It remains unclear if these patients will require a temporary or chronic VEGF suppression. Despite timely treatment with anti-VEGF agents, these eyes could potentially develop a further loss in vision and experience an increased treatment burden as well. Several hypotheses have been put forth to explain this phenomenon [34,57]. As OCTA becomes more available, it is hoped that future trials will incorporate it to further assess the mechanisms involved in the conversion of GA to exudative NV-AMD following C3 or C5 inhibition. Similarly, the unexpected findings of ION and the recent alert regarding occlusive retinal vasculitis are worrisome and merit further study [33].

In summary, the intravitreal inhibition of either C3 or C5 slows down the progression of GA. It is hoped that these results stimulate further research in the field to obtain additional insights that can fuel future drug research and development in GA and its precursor states so that patients with this affection may see some light at the end of the tunnel.

## Data Availability

Not applicable.

## Supplementary Materials

The following supporting information can be downloaded at: https://www.mdpi.com/article/10.3390/jcm12155131/s1, Table S1: Summary of the DERBY, OAKS, GATHER1 and GATHER2 Clinical Trials.
