# Supplementary material for: Complement Inhibitors for Advanced Dry Age-Related Macular Degeneration (Geographic Atrophy): Some Light at the End of the Tunnel?
Source: J Clin Med. 2023 Aug 4;12(15):5131. doi: 10.3390/jcm12155131 (PMC10420150; doi:10.3390/jcm12155131)
Supplement: Supplementary file 1 [file jcm-12-05131-s001.zip › jcm-2516323-supplementary.pdf]

Table S1. Summary of the DERBY, OAKS, GATHER 1 and GATHER 2 Clinical Trials.

	DERBY			OAKS			GATHER 1		GATHER 2	
	Peg Monthly	Peg Every Other Month	Sham	Peg Monthly (N=202)	Peg Every Other Month (N=205)	Sham	ACP 2 mg N=67	Sham N=110	ACP 2 mg N=225	Sham N=222
**Age (Years)**	78.7	79.2	78.6	78.8	78.1	78.6	78.8	78.2	76.3	76.7
**Female**	118 (58.7%)	120 (59.7%)	123 (63.1%)	125 (61.9%)	117 (57.1%)	133 (64.3%)	45 (67.2%)	79 (71.8%)	154 (68.4%)	156 (70.3)
**Baseline GA Mean Total Area (mm2)**	8.37	8.25	8.24	8.18	8.30	8.21	7.33	7.42	7.48	7.81
**Baseline Square Root GA Lesion Size (mm)**	2.80	2.79	2.78	2.78	2.80	2.79	2.62	2.63	2.64	2.71
**Baseline BCVA (Letters)**	59.5	58.7	59	61	58.2	57.6	70.2	69	70.9	71.6
**Reduction in GA Lesion Growth at Month 12 When Compared to Sham**	12% p=0.0528	11% p=0.075		22% p=0.0003	16% p=0.0052		35.4% p=0.0050		17.7% p=0.0039	
**CNV***	25 (6.0%)	17 (4.1%)	10 (2.4%)	DERBY	OAKS	COMBINED	6 (9%)	3 (2.7%)	15 (6.7%)	9 (4.1%)

	DERBY			OAKS			GATHER 1		GATHER 2	
**Endophthalmitis***	1	2	0	DERBY	OAKS	COMBINED	0	0	0	0
**IOI***	9 (2.1%)	4 (1%)	0	DERBY	OAKS	COMBINED	1 (1.5%)	0	0	0
**ION**	0	0	0	1 (0.5%)	0	0	0	0	0	0

Peg=pegcetacoplan; ACP=avacincaptad; CNV=choroidal neovascularization; IOI=intraocular inflammation; ION=ischemic optic neuropathy; * DERBY & OAKS combined
